# The *bio.tools* registry of software tools and data resources for the life sciences

**DOI:** 10.1186/s13059-019-1772-6

**Published:** 2019-08-12

**Authors:** Jon Ison, Hans Ienasescu, Piotr Chmura, Emil Rydza, Hervé Ménager, Matúš Kalaš, Veit Schwämmle, Björn Grüning, Niall Beard, Rodrigo Lopez, Severine Duvaud, Heinz Stockinger, Bengt Persson, Radka Svobodová Vařeková, Tomáš Raček, Jiří Vondrášek, Hedi Peterson, Ahto Salumets, Inge Jonassen, Rob Hooft, Tommi Nyrönen, Alfonso Valencia, Salvador Capella, Josep Gelpí, Federico Zambelli, Babis Savakis, Brane Leskošek, Kristoffer Rapacki, Christophe Blanchet, Rafael Jimenez, Arlindo Oliveira, Gert Vriend, Olivier Collin, Jacques van Helden, Peter Løngreen, Søren Brunak

**Affiliations:** 1 National Life Science Supercomputing Center, Technical University of Denmark, Building 208, DK-2800 Kongens Lyngby, Denmark; 2 Novo Nordisk Foundation Center for Protein Research, Faculty of Health and Medical Sciences, University of Copenhagen, DK-2200 Copenhagen, Denmark; 3 Hub de Bioinformatique et de Biostatistiques, Institut Pasteur, C3BI USR, 3756 IP CNRS, Paris, France; 4 Computational Biology Unit, Department of Informatics, University of Bergen, N-5020 Bergen, Norway; 5 Department of Biochemistry and Molecular Biology and VILLUM Center for Bioanalytical Sciences, University of Southern Denmark, Campusvej 55, 5230 Odense, Denmark; 6 Department of Computer Science, Albert-Ludwigs-University Freiburg, Georges-Köhler-Allee 106, 79110 Freiburg, Germany; 7 School of Computer Science, The University of Manchester, Oxford Road, Manchester, M13 9PL UK; 8 The EMBL-European Bioinformatics Institute, Wellcome Trust Genome Campus, Hinxton, Cambridge, CB10 1SD UK; 9 SIB Swiss Institute of Bioinformatics, Quartier Sorge - Batiment Amphipole, CH-1015 Lausanne, Switzerland; 10 Bioinformatics Infrastructure for Life Sciences, Science for Life Laboratory, Dept of Cell and Molecular Biology, Uppsala University, S-75124 Uppsala, Sweden; 11 CEITEC - Central European Institute of Technology, Masaryk University Brno, Kamenice 5, 625 00 Brno-Bohunice, Czech Republic; 12 Institute of Organic Chemistry and Biochemistry, Czech Academy of Sciences, Flemingovo namesti 2, 160 00 Prague, Czech Republic; 13 ELIXIR-EE, Institute of Computer Science, University of Tartu. J Liivi 2, Tartu, Estonia; 14 Dutch Techcentre for Life Sciences, Jaarbeursplein 6, 3521 AL Utrecht, The Netherlands; 15 CSC - IT Center for Science, PO BOX 405, FI-02101 Espoo, Finland; 16 Barcelona Supercomputing Centre (BSC), 08034 Barcelona, Spain; 17 Institució Catalana de Recerca i Estudis Avançats (ICREA), Pg. Lluıs Companys 23, 08010 Barcelona, Spain; 18 Department of Biochemistry and Molecular Biomedicine, University of Barcelona, INB / BSC-CNS, Barcelona, Spain; 19 Institute of Biomembranes, Bioenergetics and Molecular Biotechnologies, National Research Council (CNR), via Amendola 165/A, Bari, Italy; 20 Department of Biosciences, University of Milano, Via Celoria 26, Milan, Italy; 21 Biomedical Sciences Research Centre, Alexander Fleming 34 Al. Fleming Str, 16672 Vari, Greece; 22 Faculty of Medicine / ELIXIR-SI, University of Ljubljana, Vrazov trg 2, SI-1000 Ljubljana, Slovenia; 23 CNRS, UMS 3601, Institut Français de Bioinformatique, IFB-core, 2 rue Gaston Crémieux, F-91000 Evry, France; 24 ELIXIR-Hub, Wellcome Trust Genome Campus, Hinxton, Cambridge, CB10 1SD UK; 25 INESC-ID / Instituto Superior Técnico, R. Alves Redol 9, Lisbon, Portugal; 26 Radboud University Medical Centre, CMBI, Postbus 9101, 6500 HB Nijmegen, Netherlands; 27 Plateforme GenOuest Univ Rennes, Inria, CNRS, IRISA, F-35000 Rennes, France; 28 Aix-Marseille Univ, INSERM, lab. Theory and Approaches of Genome Complexity (TAGC), Marseille, France; 29 Department of Bio and Health Informatics, Technical University of Denmark, Building 208, DK-2800 Kongens Lyngby, Denmark

## Abstract

Bioinformaticians and biologists rely increasingly upon workflows for the flexible utilization of the many life science tools that are needed to optimally convert data into knowledge. We outline a pan-European enterprise to provide a catalogue (https://bio.tools) of tools and databases that can be used in these workflows. *bio.tools* not only lists where to find resources, but also provides a wide variety of practical information.

**Electronic supplementary material:**

The online version of this article (10.1186/s13059-019-1772-6) contains supplementary material, which is available to authorized users.

A myriad of providers - from individual scientists to large service organizations - have created thousands of databases and tools, serving a dynamic domain spanning biology, biotechnology and medicine. Scholars must contend with intrinsically complex biological data, integrated into hundreds of data formats for analysis by a vast array of methods and diverse types of software, deployments and interfaces. Developments are often ad hoc, and in the absence of a source of unified information, it is not easy to assess the scope and compatibility of new resources in context of global offerings. For example, software may lack a formalized description of its scientific and technical function, and the absence of persistent, unique tool identifiers confounds reliable citation and reproducibility of analyses. There are significant barriers to find and connect the right tools among a multitude of possibilities, making the work of the bioinformatician - developing practical workflows for scientific discovery - far from trivial.

Since the 1980s various initiatives, at a local or more global level, have catalogued bioinformatics resources to advertise their wares and guide scientists in their choices. Early single investigator initiatives include the famous Pedro’s List of weblinks and Gunnar von Heijne’s 1987 book ‘Sequence Analysis in Molecular Biology: Treasure Trove or Trivial Pursuit’ [1]. Contemporary examples include international service providers [2], laboratories (https://www.rostlab.org/), software suites (https://www.bioconductor.org/), deployment solutions [3, 4], scientific publishers [5], WIKIs such as msutils.org, and online catalogues [6] including commercial offerings such as from omicX (https://omictools.com/) and open lists such as from the BIG Data Center initiative (https://bigd.big.ac.cn/tools) based in Beijing. Such collections serve their communities well, but when taken as a corpus of information about tools in general, present a fragmented information landscape, with much redundancy. Owing to a lack of commonly adopted information standards, it can be difficult to understand what is available and compare different approaches to the same problem. Web search engines like Google provide the entry point for searches, but yield results reflecting mostly historic prevalences, insufficiently structured to allow ready comparison. Thus, in an era of highly efficient Web searches generally, barriers remain to the efficient utilization of bioinformatics resources, with continued use of suboptimal offerings, slow uptake of new tools and reinvention of existing functions.

A practical first step [7] towards a sustainable and unified resource registry engaged enthusiastic individuals from the spectrum of European bioinformatics, to share and maintain information about resources within their scope. This effort is now joined by the 22 nodes of ELIXIR (https://www.elixir-europe.org/), the European Infrastructure for Biological Information. Our aims include:

1. scientists can find, understand and compare tools for computational experiments, and access the wealth of data resources
2. bioinformaticians have clues about compatibility of tools with various data types and formats, thus, what might readily be chained into functional workflows
3. developers can find and assess implementations of desired functionality, encouraging reuse and repurposing over reinvention
4. end-users can easily find supplementary information, such as benchmarking results or training courses
5. facility managers can see the status (emerging, mature or legacy) of a resource, including licensing, and assess its applications and technical performance during service design
6. funders and reviewers have an overview of productions at various hierarchical levels such as individual, institutional or even national
7. tool developers and service providers can contribute to the registry in simple but effective ways
8. information about the legacy of resource developments does not get lost

Fulfillment of these aims requires upkeep of a high quality, non-redundant corpus of information, that is integrated with deployment solutions, scientific literature and pertinent activities including benchmarking, monitoring and training, and which can adapt to the bioinformatics landscape of tomorrow. The burden is therefore onerous. Developers and providers are best placed - and motivated - to document their own productions, but given the complex landscape they require sustained coordination and support. They have been left alone in this critical activity, and it is no surprise that a unified and enduring catalogue has remained an elusive goal. A major community-driven effort is required, sustained by long term institutional commitments. ELIXIR, as the linchpin of a network of diverse research infrastructures, is ideally positioned to promote a common strategy and deliver a portal that is broadly relevant across a range of disciplines and user groups.

Our portal (https://bio.tools), which has developed steadily over 5 years, now includes over 250,000 annotations on some 12,000 resources. All types of application software are within scope, across all life science domains globally. This includes everything from simple command-line tools and Web applications, to databases, workflows and integrated workbenches. Most entries describe open source or freely accessible tools with straightforward functions, which are therefore readily combinable into functional workflows. Accessions are assigned a unique tool identifier: a manually verified, URL-safe version of the supplied tool name. When used in combination with a version label assigned by a developer, the tool IDs provide a pragmatic means to cite and trace software, especially in the absence of a traditional publication. The IDs are used in persistent *bio.tools* URLs, resolving to Tool Cards of essential information. *bio.tools* mandates only bare-bones information (name, short description and homepage), whilst supporting rich description of 50 salient scientific, technical and administrative attributes. Resource descriptions must conform to rigorous semantics and syntax, defined in a formalized schema, biotoolsSchema (https://github.com/bio-tools/biotoolsSchema). Controlled vocabularies are used extensively, and provide concise, consistent and therefore comparable information, for the convenience of the user. For example, tools may be annotated with specific topics, operations, input and output data types and supported formats from the EDAM ontology [8]. Standard identifiers are used where possible, e.g. DOIs for publications, and verbose information, such as documentation or citation instructions, are referenced by URL. Hence, the dizzying complexity of bioinformatics software is reduced to collections of readily understandable functional units, put in scientific and technical context, including information to enable access and use. The aggregation and standardization of data under the portal can help end-users in very practical ways. Consider for example a biologist who is surveying recently published tools in a general scientific area, or for a specific computational task, and wants to identify those which are freely available for use. They can search *bio.tools* using specific EDAM topics and operations to quickly make a list of candidate tools and compare alternatives, drilling down to tools available under open license and with a recent publication. Without *bio.tools* they would need to manually search and browse a large number of web pages, ranging from software repositories (e.g. GitHub) to scientific literature resources (e.g. PubMed), which can be a time-consuming and difficult process.

The initiative upholds open science principles [9], and thus far has benefited from 1127 contributors from 422 domains. Contributions to date are mostly from Europe and the USA, which simply reflects *bio.tools’* European foundation and the high volume of American tools. There are, of course, vibrant bioinformatics communities all over the world, and we warmly welcome and encourage their participation. Direct curation assistance is available from the core *bio.tools* team, through collaboration with ELIXIR partners and at community-led workshops. The effort expected from providers is thus reduced to a relatively small and maintainable level, and we hope to attract and retain many new contributors and collaborators. Direct participation in the project and re-use of the registry is strongly encouraged. Practical information describing how scientific communities and individual software developers can contribute are available online [10, 11]. Access to the portal is unrestricted and both the registry content and portal source code (https://github.com/bio-tools/biotoolsRegistry/) are freely available under open license (CC BY 4.0 and GPL-3.0 respectively).

We have summarized our vision and progress towards a solution of a global and major challenge: a uniform means by which to describe, publish, discover and cite bioinformatics resources. *bio.tools* is a step towards a central point of unified information, to avoid the rewriting of resource descriptions in so many different contexts. The current implementation upholds the FAIR data principles [12] and, with progressive development, will help make bioinformatics resources more findable and accessible, and somewhat more interoperable and reusable. To fully realize our vision, however, involves much ongoing work:

1. inclusion of information about online services, deployment solutions and supported data formats, to provide users with information about availability and uptime, and enable tool use and applications such as automated workflow composition [13].
2. ease the curation process, e.g. by curation tools [14], and utilities [15] to pull tool information from workbench environments such as Galaxy, or, where applicable, directly from code repositories such as GitHub, and by new linting utilities (e.g. https://github.com/bio-tools/biotoolslint) to identify and fix inconsistencies in annotations.
3. leverage specialized community efforts (https://www.elixir-europe.org/communities) and biomedical science research infrastructures internationally, to expand coverage and improve quality in areas such as proteomics, metabolomics and bioimaging.
4. stable metadata sharing mechanisms for institutional collections such as IFB tools (https://www.france-bioinformatique.fr/en/services/tools) and specialized registries such as BioContainers [16].
5. inclusion of Web APIs and services for accessing the multitude of biological databases, e.g. by developing systems [17] that leverage community standards such as OpenAPI (https://www.openapis.org).
6. expose quality metrics to provide a trustworthy and rational means for tool assessment, including scientific benchmarking of analytical tools and monitoring of service technical robustness, from platforms such as ELIXIR openEBench (https://openebench.bsc.es).
7. services [18] to combine and export *bio.tools* data with execution-layer information in specific workflow configuration formats such as used by Galaxy [19] or a generic one such as the Common Workflow Language (https://www.commonwl.org/).
8. more convenient and powerful interfaces and features for query formulation, searching and browsing.
9. enhancing the management of user profiles and crediting of contributions, e.g. using ELIXIR AAI [20] federated user identity management, which incorporates researcher identities such as ORCID (https://orcid.org/)
10. crosslink with portals such as ELIXIR TeSS [21] (training resources) and FAIRSharing [22] (data standards), in order to make navigation of the broader bioinformatics resource landscape more coherent and convenient

With community support, *bio.tools* can become a standard way to disseminate publicly-funded software development. The primary long-term challenge is to nurture the community around it and ensure the portal matches end-user requirements. Here, the anchoring within ELIXIR allows us to draw upon a coordinated, European-wide community of experts, including national service managers. Long-term support from these partners, and synergistic relationships with community projects and other major international initiatives, will sustain the portal in the long term, allowing for secure planning and investment. We welcome collaborations with all scholars on common goals, and encourage life scientists worldwide to join forces in a task that can greatly benefit the whole community.

## Additional file

Additional file 1: Review history. (DOCX 20 kb)

## Data Availability

The *bio.tools* content is freely available to all via the *bio.tools* API under the Creative Commons Attribution license (CC BY 4.0). The source code of the registry is available under standard GPL 3.0 license from GitHub (https://github.com/bio-tools/biotoolsRegistry/) [23]. EDAM and biotoolsSchema are licensed under the Creative Commons Attribution-ShareAlike 4.0 International License (CC BY-SA 4.0).
