# Supplementary material for: The bio.tools registry of software tools and data resources for the life sciences
Source: Genome Biol. 2019 Aug 12;20:164. doi: 10.1186/s13059-019-1772-6 (PMC6691543; doi:10.1186/s13059-019-1772-6)
Supplement: Supplementary file 1 — Review history. (DOCX 20 kb) [file 13059_2019_1772_MOESM1_ESM.docx]

Review History

**First round of review**

**Reviewer 1**

**Are you able to assess all statistics in the manuscript, including the appropriateness of statistical tests used?**

There are no statistics in the manuscript.

**Comments to author:**

In the open letter "The bio.tools registry of software tools and data resources for the life sciences" Jon Ison et al summarize the current state, goals, and vision of Bio.tools. Bio.tools is an open and well-established repository of tools, databases, and services for bioinformatics and other life sciences containing more than 12 thousand entries from thousands of contributors. The open letter is concise, well-written and has a clear message of inviting the community to further contribute to the expansion and comprehensiveness of the open catalog. We suggest the letter to be accepted with minor changes.

Overall, I believe that having central repositories is an excellent way forward for the genomics community. For example, genomics has experienced the concentration of data in large, central and public repositories from NIH and EBI. The early adoption of these repositories has driven bioinformatics to develop a concise community, which adopts a relatively small number of standards and file formats. Perhaps, having a central repository for applications could push the genomics community to create better code, better documentation, and more mature bioinformatics pipelines. Bio.tools might fill the gap central repositories for bioinformatics (and life-science tools).

Minor comments:
Line 20: From the manuscript, but in particular in this line, it is clear that the developers of Bio.tools employed a great effort in building an open platform with open and public API and database schemes. These topics are of great value to bioinformaticians and computer scientists who are planning to, for example, mine the bio.tools metadata. While these are valuable and should remain in the letter, this text implicitly suggests that the main users bio.tools are perhaps bioinformatics and computer scientists. Nonetheless, some biologists, who might, in fact, comprise a larger component of the community, and may be less versed in computational frameworks are also potentially big beneficiaries of bio.tools. Addressing the benefits for those users could promote this letter to target a larger audience. 

Line 29: In addition to the cited alternatives to bio.tools, Omic.tools and bigd.big.ac.cn could be also be cited as an alternative resource for genomics tools and databases contributing to the fragmentation of the community.

Line 30: The sentence "present a fragmented information landscape with much redundancy" is vague and somewhat unclear. Perhaps showing evidence that this is the case could advocate a larger for the adoption of bio.tools.

Line 27: Similar to the criticism in Line 20, further exploring the benefits of the bio.tools to biologists would expand the target base for the catalog. 

Line 57: The authors should be aware and possibly explicit on the biases for European and American resources. Bio.tools could also be an interesting venue for regions with smaller but still vibrant bioinformatics communities such as Africa and South America. 

* It is somewhat sticking that resources such as GENCODE, one of the main resources of annotation of the human genome, are not yet described in bio.tools. Perhaps leveraging this exposure to the genomics community to include most of these well-established annotations, databases, and tools in the bio.tools catalog is a way forward.

**Reviewer 2**

**Are you able to assess all statistics in the manuscript, including the appropriateness of statistical tests used?**

There are no statistics in the manuscript.

**Comments to author:**

With this presentation of bio.tools, Ison and colleagues address an important bioinformatics challenge that is timely, and well resourced on that it will take the entire community to address and to manage. The problem of finding the best resource to do the job you need to perform, in the context that suits ones specific needs is a laudable task, and these authors have developed a tool that will provide its users the right tool to achieve this. 

That said, there are a number of challenges that do present themselves that should be stated up front. There are challenges with complexity, uniformity, tanking of results and expectations.

The bio.tools has a intuitive user interface, and has data you expect to see in such a resource. The way ontologies and the curation mechanism is used make it rich and important resource to the community. One bias it does appear to show is that it partial to European tools, or even European version of tools developed elsewhere. I am just stating that there might be a bias, and that this can easily be corrected by stating up front that this is and that the world community is invited to correct this, They do mention in the last sentence " … and encourage life scientists worldwide to join forces in a task that can greatly benefit the whole community.", without mentioning that they know they have a bias.

A challenge with tools like "catalogues" of everything where you want a presentations of "hits" you are looking for to be organized a certain way, and when you don't see that you then try to understand about why is the tool not presenting the things you expect to see. To explain this better, I will present one example of a search I did on bio.tools, and ask the authors to maybe address or explain why I have the results I see.

Let us start with the search for "BLAST" and select the suggested result "BLAST"

Page 1:

BLAST | 
blastprodom
GHOSTZ | 
BLAST2SRS
Blast2GO | 
H-BLAST | 
QSCOP BLAST | 
G-BLASTN | 
BWT-SW | 
BLASTO

Page 2:

TARA BLAST Service
BLAST API (EBI) | 
BlastR | 
BTOD | 
PSI-BLAST API (EBI) | 
Orthologue Search Service | 
MPBLAST | 
Bujnicki Lab Tools | 
MS-BLAST | 
HS-BLASTN | 

Page 3:

PDB-BLAST | 
FastHMM | 
BLAST (EBI) | 
BLAST (SIB) | 
PSI-BLAST (EBI) | 
SequenceServer | 
ChEMBL BLAST Search | 
Ensembl Genomes Plants Blast | 
Ensembl Sequence Search BLAST | 


Although I understand the fact that NCBI's BLAST server is the first one on this list, 
I don't understand why the BLAST (EBI) server (or the SIB one) is not 2nd and third, 
but rather 2 pages down? Incidentally, the BLAST from NCBI (to be consistent with 
the others), should be labelled "BLAST (NCBI)". Also, bio.tools indicates that NCBI 
BLAST is only Linux and Mac, which is wrong, as they also offer windows binaries, 
see here: https://ftp.ncbi.nih.gov/blast/executables/LATEST/

Also, if you look at the tagging of those three: BLAST (NCBI), BLAST (EBI) and 
BLAST (SIB)

You have:

BLAST | 
Sequence analysis
Molecular genetics
Statistics and probability
Genomics
Sequence similarity search 
Sequence alignment
Command-line tool
Web service

BLAST (SIB) | 
Proteomics
Sequence similarity search 
Sequence alignment
Web application

BLAST (EBI) | 

Sequence analysis
Sequence similarity search 
Sequence alignment
Command-line tool
Web application


Although I appreciate BLAST (SIB) may not support the command line tool, 
all other tags should be the same, and SIB even offers blastn of nucleotides 
query against a nucleotide DB!

I think all of this is inconsistent , only one example (although I would suggest 
that "blast" is a good test case, and one could nit-pick across other tools. 
For example the entry for BWA or BWAmem (absent), or SamTools is pretty 
weak, although good for GATK.

So what I would suggest you do is present more on how the community can 
help. I think it is one thing to invite tool developers to maintain there tools 
(and I have ideas how to maintain this), but there needs to be a system to 
catch and quickly correct inconsistencies, and I think this communication 
should talk a little bit more about that. 


Minor concerns that should maybe addressed:

I would have really liked a summary of my hits, so I can look and see what there is, the "Compact form" is not compact enough. I would like to see one line per hit. Also, would be useful to change the number of hits you see per page, 10, 50, 100, 200?

As a bio.tools users (and wanting to do different searches, and compare results etc), it would be useful to be able to download my results, may maybe into a CSV or TSV format, with all fields you maintain?

I would have also liked to see a definition of "score", maybe how these are calculated, and what the scores are. Maybe two items result from a search have a very similar score (I will look at both), or are very far from each other (I will look at one 1st).

The authors mention: "Most entries describe open-access tools with straightforward functions"

"Open Access" is usually used to describe publications, and I think that when talking about tools (software), one usually refers to them as "Open Source". That said, I have seen "Open Access" used for databases as well.


The authors mention: "the dizzying complexity of bioinformatics software". Maybe a better term to describe this complexity could be used? "Vast"? "Astronomic"? "Epic"?

Otherwise, a well written communication, that talk about a tool that needs to be used by the community,

@bffo

--
BF Francis Ouellette                     [francis.ouellette@gmail.com](mailto:francis.ouellette@gmail.com)

**Authors Response**

Reviewer 1 report

“Line 20: … Nonetheless, some biologists, who might, in fact, comprise a larger component of the community, and may be less versed in computational frameworks are also potentially big beneficiaries of bio.tools. Addressing the benefits for those users could promote this letter to target a larger audience.”

“Similar to the criticism in Line 20, further exploring the benefits of the bio.tools to biologists would expand the target base for the catalog.”

*bio.tools is indeed built to cater for the needs of biologists and other roles, in addition to bioinformaticians, which we’d listed after “Our aims include:” We have added a few sentences illustrating the benefits specifically to biologists (at the end of the paragraph beginning “Our portal …”).*

“In addition to the cited alternatives to bio.tools, Omic.tools and bigd.big.ac.cn could be also be cited as an alternative resource for genomics tools and databases contributing to the fragmentation of the community.”

*There are many such resources on the web. We’ve added links for omictools.com (a commercial site with closed data and closed source) and bigd.big.ac.cn (essentially a list of tool names with descriptions), to serve as additional examples.*

“Line 30: The sentence "present a fragmented information landscape with much redundancy" is vague and somewhat unclear. Perhaps showing evidence that this is the case could advocate a larger for the adoption of bio.tools.”

*The point here is that historically there have been very many different initiatives and efforts, often with overlapping content, and adhering to different (or usually no) information standards We have reworked the sentence to make the meaning more clear.*

*Line 57: The authors should be aware and possibly explicit on the biases for European and American resources. Bio.tools could also be an interesting venue for regions with smaller but still vibrant bioinformatics communities such as Africa and South America.*

*We have added a couple of sentences (to the paragraph beginning “The initiative upholds …”) to explain why bio.tools contains mostly European and American software but warmly welcomes and supports contributions globally. Our initial efforts focused on ELIXIR (European) resources, and there is simply more software originating from Europe and America than the rest of the world. But we do include tools from all over the world; it’s hard to calculate the geographical origin of the tools precisely but a quick analysis of top-level domains of tool homepages shows, for example, 3820, 1900 and 565 tools from “.org”, “.com” and “.net” domains respectively (which are often international), 296 from China, 185 from Canada, 160 from Japan, 142 from India and 118 from Taiwan.*

“It is somewhat sticking that resources such as GENCODE, one of the main resources of annotation of the human genome, are not yet described in bio.tools. Perhaps leveraging this exposure to the genomics community to include most of these well-established annotations, databases, and tools in the bio.tools catalog is a way forward.”

*We have added the GENCODE portal to bio.tools, and always try promptly to respond to such suggestions. We do indeed hope to reach a broader audience with this article, and get many more contributions to help fill in any gaps. We also have utilities in development to more systematically mine the literature (past and new articles) for relevant software; these are not mentioned explicitly in the article but see* [*https://github.com/edamontology/edammap*](https://github.com/edamontology/edammap) *and* [*https://github.com/bio-tools/pub2tools*](https://github.com/bio-tools/pub2tools)*.*

Reviewer 2 report

“One bias it does appear to show is that it partial to European tools, or even European version of tools developed elsewhere. I am just stating that there might be a bias, and that this can easily be corrected by stating up front that this is and that the world community is invited to correct this, They do mention in the last sentence " … and encourage life scientists worldwide to join forces in a task that can greatly benefit the whole community.", without mentioning that they know they have a bias.

*Reviewer 1 also raised this issue, which we have addressed in the manuscript (please see “Reviewer 1 report” above)*

“A challenge with tools like "catalogues" of everything where you want a presentations of "hits" you are looking for to be organized a certain way, and when you don't see that you then try to understand about why is the tool not presenting the things you expect to see. ….. Although I understand the fact that NCBI's BLAST server is the first one on this list, I don't understand why the BLAST (EBI) server (or the SIB one) is not 2nd and third, but rather 2 pages down?”

*This is indeed a challenging issue, as people - naturally - tend to compare search performance nowadays to the major search engines such as Google (which of course have massive investment). We have performed several rounds of tweaking the search behaviour in light of real user testing, and it is becoming progressively more intuitive, but this is also not trivial as each type of user has different sets of expectations. The case in point (“BLAST” example) is there are many entries which match “blast” both in name and / or short description, which are then rendered as top-scoring hits but in no particular order, other than “BLAST” appearing first because of the exact full-length match to the name. In light of this feedback, we are exploring how to optimise the search behaviour for this specific use-case.*

“Incidentally, the BLAST from NCBI (to be consistent with the others), should be labelled "BLAST (NCBI)".”

*NCBI BLAST has the tool ID of “blast” and is simply called “BLAST” because it represents the “canonical” BLAST entry, i.e. NCBI are providers of the original BLAST tool on which all the others depend. So in this case, we think the name and ID is OK (see point below re consistency of annotations for context).*

“Also, bio.tools indicates that NCBI BLAST is only Linux and Mac, which is wrong, as they also offer windows binaries, see here: <https://ftp.ncbi.nih.gov/blast/executables/LATEST/>

*The “Windows” operating system annotation has been added to the entry.*

“Also, if you look at the tagging of those three: BLAST (NCBI), BLAST (EBI) and BLAST (SIB) ….. I think all of this is inconsistent , only one example (although I would suggest that "blast" is a good test case, and one could nit-pick across other tools.”

*The BLAST example points to the challenges faced in producing bio.tools. While the functional annotation (inputs, operations and outputs) were in fact consistent across BLAST (NCBI), BLAST (SIB) and BLAST (EBI) some of the miscellaneous other annotations were not, as the reviewer indicates. Notably the “topics” (which have been cleaned up and are now consistent) and the “tool types” (which now accurately and comprehensively represent what is actually provided at each site). The inconsistencies arise because the entries are independently edited, but with central curation to clean things up, as soon as we’re alerted to the problems (as the case here).*

*These entries are examples of where one underlying tool (BLAST) is served up in different places, with slightly different end-user options. bio.tools tries, and mostly succeeds, in rendering unique “canonical” entries / tool descriptions. In this case, NCBI BLAST is the “canonical” tool and the others provide access over it. We’re exploring ways to handle this better, technically, to improve consistency, perhaps aiming for a single entry (with the basic tool description) and then a section for various interfaces / access points.*

*To those ends, we’ve implemented a basic entry relationship model in bio.tools which allows one to define relationships, e.g. BLAST (SIB) uses BLAST, which you can see e.g. in* [*https://bio.tools/blast_ebi*](https://bio.tools/blast_ebi) *and various entries under* [*http://proteomics.bio.tools*](http://proteomics.bio.tools/)*. We’re in the process of curating such relationships, and this will help to consolidate the content in due course - this is tricky work because one needs to make a judgement call as to what constitutes a unique tool, and what is the “canonical” entry!*

“So what I would suggest you do is present more on how the community can help.”

*We recently had accepted for publication in Briefings in Bioinformatics an article that explains, in practical details, how an individual or community can contribute to bio.tools. That article goes into a lot of technical details which are definitely out of scope (and complement) this Open Letter. We now have a DOI for the BiB article and have cited it. We also include a link to our online documentation - which includes the bio.tools Contributors Guide and Curators Guide, and is a comprehensive source of practical help.*

“I think it is one thing to invite tool developers to maintain there tools (and I have ideas how to maintain this), but there needs to be a system to catch and quickly correct inconsistencies, and I think this communication should talk a little bit more about that.”

*The adoption of bio.tools entries by developers is the best hope to drive quality in the long-term. bio.tools has seen strong and consistent growth in contributors since the outset, but we’re very open to explore new ways to promote adoption and would like to learn more about the reviewers ideas (please ask them to get in touch directly). One option is to identify and directly contact prospective owners of “orphan” entries, i.e. tools which were added to bio.tools but not by the developer themselves. To that end, as an experiment, we have contacted all the prospective entry owners of “orphan” tools in the proteomics tool subset (*[*https://proteomics.bio.tools*](https://proteomics.bio.tools/)*) suggesting they adopt the entries. From the responses we have to date, this is looking like a good way to solicit contributions.*

*As for catching and correcting inconsistencies, a technical prerequisite is knowledge of related entries, hence we implemented the tool relationship model summarised above. Consolidation of entries, where possible, would remove some inconsistencies, and in principle the remaining could be identified (given accurately defined tool relationships) in an automatic way. Such functionality could be worked into the “linting” tool (*[*https://github.com/bio-tools/biotoolslint*](https://github.com/bio-tools/biotoolslint)*) that we are currently specifying (and this issue is now specifically tracked at* [*https://github.com/bio-tools/biotoolsLint/issues/49*](https://github.com/bio-tools/biotoolsLint/issues/49)*).*

*We have revised point 2 of the list of future work to include this aspect, but would prefer to keep it to that, to avoid a more lengthy description of technical details.*

“I would have really liked a summary of my hits, so I can look and see what there is, the "Compact form" is not compact enough. I would like to see one line per hit.”

*This is a sensible request which has been made by others in the past (see* [*https://github.com/bio-tools/biotoolsRegistry/issues/381*](https://github.com/bio-tools/biotoolsRegistry/issues/381)*) and will be acted on in due course.*

“Also, would be useful to change the number of hits you see per page, 10, 50, 100, 200?”

*We will add new paging options for 10, 25, 50 and 100 hits in due course - a feature which has been requested by others (see* [*https://github.com/bio-tools/biotoolsRegistry/issues/325*](https://github.com/bio-tools/biotoolsRegistry/issues/325)*).*

“As a bio.tools users (and wanting to do different searches, and compare results etc), it would be useful to be able to download my results, may maybe into a CSV or TSV format, with all fields you maintain?”

*This is a reasonable request but may not be practical for all the data, given that some of it (e.g. tool function information) is structured and so not very amenable to dumping in CSV format. We’ve logged the issue (*[*https://github.com/bio-tools/biotoolsRegistry/issues/451*](https://github.com/bio-tools/biotoolsRegistry/issues/451)*) and will act on it in due course.*

“I would have also liked to see a definition of "score", maybe how these are calculated, and what the scores are. Maybe two items result from a search have a very similar score (I will look at both), or are very far from each other (I will look at one 1st).”

*This has been requested by others in the past, but would be very difficult to describe in an intuitive way, as it depends on the intricacies of the elasticsearch indexing / scoring method. For now, we have not taken action.*

“The authors mention: "Most entries describe open-access tools with straightforward functions". "Open Access" is usually used to describe publications, and I think that when talking about tools (software), one usually refers to them as "Open Source". That said, I have seen "Open Access" used for databases as well.”

*We have revised the text to say “open source or freely accessible” instead of “open-access”, which we define to have a specific meaning concerning online services (see* [*https://biotools.readthedocs.io/en/latest/curators_guide.html#accessibility*](https://biotools.readthedocs.io/en/latest/curators_guide.html#accessibility)*)*

The authors mention: "the dizzying complexity of bioinformatics software". Maybe a better term to describe this complexity could be used? "Vast"? "Astronomic"? "Epic"?

*We’ve dropped the use of “dizzying”*
